# One-year risk of serious infection in patients treated with certolizumab pegol as compared with other TNF inhibitors in a real-world setting: data from a national U.S. rheumatoid arthritis registry

**DOI:** 10.1186/s13075-017-1496-5

**Published:** 2018-01-02

**Authors:** Leslie R. Harrold, Heather J. Litman, Katherine C. Saunders, Kimberly J. Dandreo, Bernice Gershenson, Jeffrey D. Greenberg, Robert Low, Jeffrey Stark, Robert Suruki, Srihari Jaganathan, Joel M. Kremer, Mohamed Yassine

**Affiliations:** 1 University of Massachusetts Medical School, Worcester, MA USA; 2 Pharmacoepidemiology and Outcomes Research, Corrona, 352 Turnpike Road, Suite 325, Southborough, MA 01772 USA; 3 New York University School of Medicine, New York, NY USA; 4 UCB Pharma, Smyrna, GA USA; 5 UCB Pharma, Raleigh, NC USA; 6 Albany Medical College, Albany, NY USA

**Keywords:** Rheumatoid arthritis, TNF inhibitors, Serious infection, Malignancy, Cardiovascular events

## Abstract

**Background:**

Registry studies provide a valuable source of comparative safety data for tumor necrosis factor inhibitors (TNFi) used in rheumatoid arthritis (RA), but they are subject to channeling bias. Comparing safety outcomes without accounting for channeling bias can lead to inaccurate comparisons between TNFi prescribed at different stages of the disease. In the present study, we examined the incidence of serious infection and other adverse events during certolizumab pegol (CZP) use vs other TNFi in a U.S. RA cohort before and after using a methodological approach to minimize channeling bias.

**Methods:**

Patients with RA enrolled in the Corrona registry, aged ≥ 18 years, initiating CZP or other TNFi (etanercept, adalimumab, golimumab, or infliximab) after May 1, 2009 (*n* = 6215 initiations), were followed for ≤ 12 months. A propensity score (PS) model was used to control for baseline characteristics associated with the probability of receiving CZP vs other TNFi. Incidence rate ratios (IRRs) of serious infectious events (SIEs), malignancies, and cardiovascular events (CVEs) in the CZP group vs other TNFi group were calculated with 95% CIs, before and after PS matching.

**Results:**

Patients were more likely to initiate CZP later in the course of therapy than those initiating other TNFi. CZP initiators (*n* = 975) were older and had longer disease duration, more active disease, and greater disability than other TNFi initiators (*n* = 5240). After PS matching, there were no clinically important differences between CZP (*n* = 952) and other TNFi (*n* = 952). Before PS matching, CZP was associated with a greater incidence of SIEs (IRR 1.53 [95% CI 1.13, 2.05]). The risk of SIEs was not different between groups after PS matching (IRR 1.26 [95% CI 0.84, 1.90]). The 95% CI of the IRRs for malignancies or CVEs included unity, regardless of PS matching, suggesting no difference in risk between CZP and other TNFi.

**Conclusions:**

After using PS matching to minimize channeling bias and compare patients with a similar likelihood of receiving CZP or other TNFi, the 1-year risk of SIEs, malignancies, and CVEs was not distinguishable between the two groups.

**Electronic supplementary material:**

The online version of this article (doi:10.1186/s13075-017-1496-5) contains supplementary material, which is available to authorized users.

## Background

Rheumatoid arthritis (RA) is a chronic autoimmune disease that causes persistent synovial inflammation. When not treated adequately, active RA can lead to progressive joint damage, significant pain, disability, and reduced quality of life [1, 2]. The chronic inflammation inherent to RA has consequences that go beyond the damage to the musculoskeletal system. Compared with the general population, RA is associated with substantial morbidity and premature mortality, which is attributed mainly to serious infection, cardiovascular disease, and certain types of cancer [3–5]. The substantial disease burden of RA underscores the importance of effective therapy.

Tumor necrosis factor inhibitors (TNFi), often the first class of biologic therapy prescribed to patients with RA, have been demonstrated to reduce disease activity and improve clinical, radiographic, and functional outcomes [6]. Effective control of disease activity with TNFi has been linked with a reduced risk of cardiovascular disease [7, 8]. Conversely, the immunosuppressive effect of TNFi has raised concerns over an increased risk of infection [9], although the magnitude of this risk remains a topic of intense debate [10–21]. The impact of TNFi on the incidence of cancer is also unclear [11, 22–25].

Overall, it is difficult to assess the risks associated with TNFi therapy, owing to the influence of patients’ demographic characteristics, clinical history, and concomitant immunomodulatory treatments. Furthermore, in clinical practice, drugs with similar therapeutic indications can be prescribed selectively to patients with different baseline prognoses, a phenomenon termed *channeling bias* [26]. Consequently, the line of TNFi therapy may also influence the safety risks observed in clinical practice.

Certolizumab pegol (CZP), a PEGylated, Fc-free TNFi, is approved for the treatment of adult patients with moderate to severe active RA [27]. Currently, there is limited evidence on the safety of CZP compared with other TNFi drugs in the context of U.S. clinical practice. The objective of this prospective, observational cohort study was to examine the 1-year incidence of serious infectious events (SIEs) during CZP use compared with other TNFi drugs (golimumab, etanercept, adalimumab, and infliximab), with and without a methodological approach accounting for channeling bias in patients with moderate to severe RA enrolled in the Consortium of Rheumatology Researchers of North America (Corrona) registry. The 1-year risk of malignancies and cardiovascular events (CVEs) was also assessed, owing to their importance for decision-making in clinical practice.

## Methods

### Data source

The Corrona registry is an independent, prospective, observational cohort of patients with RA recruited from 169 private and academic practice sites across 40 states in the United States [28]. Data on 43,099 patients with RA had been collected as of June 30, 2016. The Corrona database comprises information from 326,613 patient visits and approximately 145,526.5 patient-years (PY) of total follow-up, with a mean patient follow-up of 4.13 years, and median time between follow-up visits of 4.90 months. Institutional review board (IRB) approvals for this study were obtained from a central IRB (New England IRB) for private practice sites and local IRBs of participating academic sites.

### Study population

Data were provided by treating rheumatologists for patients with RA enrolled in the Corrona registry who initiated treatment with CZP or other TNFi (adalimumab, etanercept, golimumab, and infliximab) between May 1, 2009, and March 31, 2016. Patients could have been treated with TNFi before this study, so index drug corresponded to any line of therapy. If patients were treated with more than one TNFi during the study, all TNFi initiations were included in the analysis. The study population comprised patients aged ≥ 18 years with at least one follow-up visit post-drug initiation. All patients provided written informed consent prior to participation.

### Adverse events of interest

Physician-reported adverse events (AEs) of interest that occurred from drug initiation up to 90 days following discontinuation/switch of TNFi, or up to 12 months from drug initiation, were included in the analysis. SIEs were the main AE of interest (infections requiring hospitalization and/or intravenous antibiotics); when data were available, information was also provided about the SIE microorganism (opportunistic vs nonopportunistic), malignancies, and CVEs (Table 1).

**Table 1:** Adverse events of interest

AE category	Physician-reported AEs included in the analysis
SIEs	Infections for which the patient was hospitalized and/or received intravenous antibiotics, which were categorized as follows: joint/bursa, cellulitis/skin, sinusitis, diverticulitis, sepsis, pneumonia, bronchitis, gastroenteritis, meningitis/encephalitis, urinary tract infection, upper respiratory infection, active tuberculosis (latent tuberculosis infection was not included), and other infections
SIE microorganism	Opportunistic infections: coccidioidomycosis, *Cryptococcus*, herpes zoster, histoplasmosis, John Cunningham virus, *Listeria*, *Pneumocystis*, active tuberculosis (latent tuberculosis infection was not included), and other opportunistic infections. Nonopportunistic infections: methicillin-resistant *Staphylococcus aureus* (MRSA), bacterial infection other than MRSA, and other nonopportunistic infections
Malignancies	Nonmelanoma skin cancer, melanoma skin cancer, lymphoma, breast cancer, lung cancer, and other cancers
CVEs	Myocardial infarction, transient ischemic attack, stroke, congestive heart failure with hospitalization, cardiac revascularization procedure, ventricular arrhythmia, cardiac arrest, acute coronary syndrome, unstable angina, hypertension with hospitalization, peripheral arterial thromboembolic event, urgent peripheral arterial revascularization, peripheral ischemia or gangrene (necrosis), and other CVEs

*Abbreviations: AE* Adverse event, *SIE* Serious infection event, *CVE* Cardiovascular event

Other AEs of interest included anaphylaxis/allergic reaction, drug-induced systemic lupus erythematosus, gastrointestinal perforation, hepatic events, progressive multifocal leukoencephalopathy, other neurological events with hospitalization and/or other demyelinating disease, and spontaneous serious bleeding (*see* Additional file 1: Table S1). Corrona has an established system for the validation of physician-reported AEs. Briefly, serious AEs and AEs of special interest are recorded by treating physicians using Targeted Adverse Event questionnaires. These questionnaires, alongside supporting documents appropriate to the event (e.g., hospitalization records, pathology reports), are submitted to Corrona for validation, with a subset triaged for expert adjudication. Previous validation of Corrona’s AE reporting has found positive predictive values of 86% for malignancies [29], 96% for CVEs [30], and 71% for SIEs [31].

### Propensity score matching

To control for baseline patient characteristics associated with the likelihood of receiving CZP or an alternative TNFi, a propensity score (PS; i.e., the probability of treatment selection) was calculated for each patient using a logistic regression model that included baseline covariates with *p* < 0.1 for the difference between CZP and other TNFi and thought to be independently associated with treatment on the basis of clinical expertise and prior group consensus. CZP and other TNFi patients were matched by estimated PS from a model including age, sex, disease duration, Clinical Disease Activity Index (CDAI) at drug initiation, and physician-reported line of TNFi therapy. Matching was performed in a 1:1 ratio without replacement, using a maximum tolerated difference (caliper) of 0.01.

### Statistical analysis

Patients’ comorbidity scores were calculated using a modified version of the Charlson comorbidity index [32] (*see* Additional file 1). All patients had a modified Charlson comorbidity index ≥ 1 because RA is included under connective tissue disease.

Baseline characteristics were compared between CZP and other TNFi patients before and after PS matching. Student’s two-sample *t* tests were used for continuous variables (or Wilcoxon rank-sum tests if variables were skewed), and chi-square tests were used for categorical variables (or Fisher’s exact test in the case of small counts). All *p* values were nominal in nature and should be interpreted in an exploratory manner.

Observed incidence rates (IRs) of AEs of interest were calculated per 100 PY, with 95% CIs calculated using the Poisson distribution assumption. For each AE category, time at risk was measured from drug initiation up to either the occurrence of the first event of interest under that category, 90 days following discontinuation/switch of biologic (censored), or up to 12 months after drug initiation (censored). Incidence rate ratios (IRRs) were calculated in both unmatched and PS-matched comparisons for overall SIEs, malignancies, and CVEs, with CZP as numerator and other TNFi as denominator; 95% CIs were calculated using the exact distribution derived using Stata® version 14.1 software (StataCorp LP, College Station, TX, USA).

## Results

### Baseline demographics

**Fig. 1:**
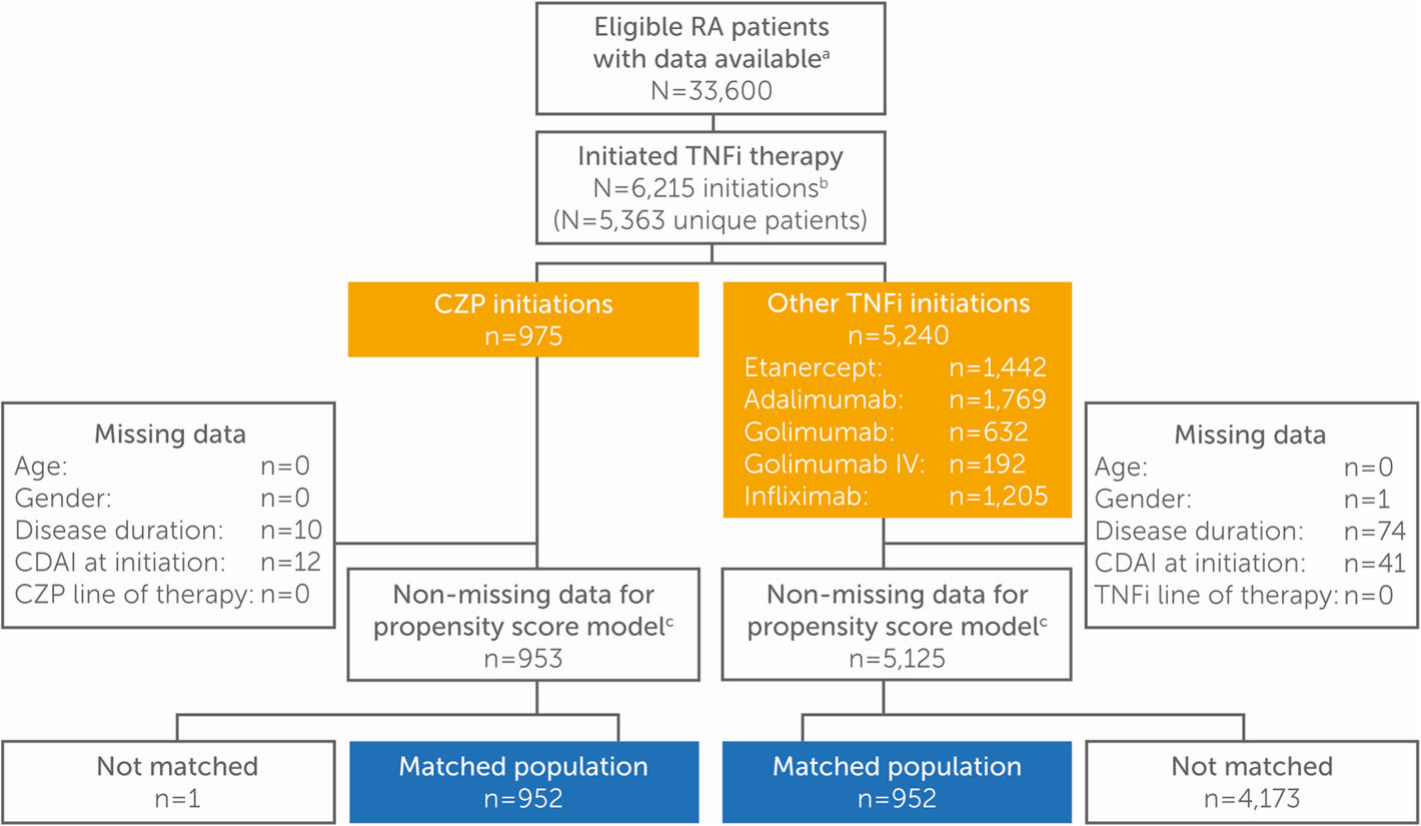
Patient disposition. Data from May 1, 2009, through March 31, 2016, were available. There were 975 unique CZP patients and 4625 unique other TNFi patients. ^a^Eligible patients with RA were ≥ 18 years of age, had at least one follow-up visit post-index drug initiation, and provided written informed consent prior to participation. ^b^Initiations refer to the total number of TNFi initiated during the period of observation because patients may have used more than one TNFi during the study (e.g., if one patient switched from etanercept to CZP, both initiations would have been included in the analysis). ^c^Propensity scores were calculated using a logistic regression model fitted with the following baseline covariates: age, sex, disease duration, CDAI at initiation, and TNFi line of therapy. *RA* rheumatoid arthritis, *CZP* certolizumab pegol, *TNFi* tumor necrosis factor inhibitor, *IV* intravenous, *CDAI* Clinical Disease Activity Index

A total of 5363 patients with RA in the Corrona database met the inclusion criteria and initiated TNFi therapy between May 1, 2009, and March 31, 2016. There were a total of 6215 TNFi initiations comprising 975 CZP initiators and 5240 initiators of other TNFi (adalimumab, etanercept, golimumab, and infliximab). After PS matching, the CZP and other TNFi groups included 952 patients each (Fig. 1).

Among all initiators (before PS matching), on average, patients initiating CZP were older, had longer disease duration, had more active disease based on the CDAI, and had more functional impairment based on the modified Health Assessment Questionnaire than patients in the other TNFi group (*p* ≤ 0.01) (Table 2). Race and Medicare insurance status also showed an imbalance between the two groups (*p* < 0.001). Sex, body mass index (BMI), age at RA onset, and the prevalence of comorbidities were balanced between groups (Table 2), as were rheumatoid factor positivity, anticitrullinated protein antibody positivity, smoking status, and blood pressure (not shown). Patients were more likely to initiate CZP later in the course of therapy, with approximately 44.8% of CZP initiators corresponding to third-line therapy or later, compared with 20.1% in the other TNFi group (Table 2). Concomitant disease-modifying antirheumatic drug (DMARD) and prednisone use were similar between the two groups, except for methotrexate (MTX) and other nonbiologic DMARD use, which was more prevalent among other TNFi initiators (Table 2).

**Table 2:** Baseline demographics, disease characteristics, tumor necrosis factor inhibitor line of therapy, and concomitant medications, before and after propensity score matching

	Before PS matching			After PS matching		
	CZP (*n* = 975)	Other TNFi (*n* = 5240)	Nominal *p* value	CZP (*n* = 952)	Other TNFi (*n* = 952)	Nominal *p* value
Demographics						
Age, years, median (IQR)	58 (18.0)	56 (16.5)	< 0.001	58 (17.0)	58 (16.0)	0.48
Female sex, *n* (%)	769 (78.9)	4107 (78.4)	0.74	751 (78.9)	750 (78.8)	0.96
Race, white, *n* (%)	840 (86.2)	4310 (82.3)	< 0.001	820 (86.1)	786 (82.6)	0.03^a^
BMI, kg/m^2^, mean (SD)	30.0 (7.3)	30.1 (7.3)	0.49	30.0 (7.3)	30.1 (7.5)	0.68
Insurance^b^						
Medicare, *n* (%)	321 (32.9)	1363 (26.0)	< 0.001	315 (33.1)	293 (30.8)	0.28
Medicaid, *n* (%)	48 (4.9)	327 (6.2)	0.12	48 (5.0)	59 (6.2)	0.32
Private, *n* (%)	712 (73.0)	4034 (77.0)	0.01	700 (73.5)	719 (75.5)	0.32
None, *n* (%)	23 (2.4)	136 (2.6)	0.74	22 (2.3)	14 (1.5)	0.24
Clinical characteristics						
Age at RA onset, years, mean (SD)	46.9 (14.4)	47.4 (13.5)	0.35	47.0 (14.4)	46.7 (13.8)	0.73
Disease duration, years, median (IQR)	9 (12.0)	5 (10.0)	< 0.001	9 (12.0)	8 (12.5)	0.26
CDAI, median (IQR)	19.0 (20.2)	17.0 (19.0)	0.01	19.0 (20.2)	18.5 (20.0)	> 0.99
mHAQ, median (IQR)	0.5 (0.8)	0.4 (0.8)	< 0.001	0.5 (0.8)	0.5 (0.8)	0.89
History of comorbidities						
Diabetes, *n* (%)	75 (7.7)	467 (8.9)	0.22	75 (7.9)	96 (10.1)	0.09
Pulmonary disease,^c^ *n* (%)	58 (5.9)	347 (6.6)	0.43	58 (6.1)	64 (6.7)	0.57
Cardiovascular disease,^d^ *n* (%)	57 (5.8)	283 (5.4)	0.57	54 (5.7)	57 (6.0)	0.77
Malignancy,^e^ *n* (%)	48 (4.9)	225 (4.3)	0.38	46 (4.8)	45 (4.7)	0.91
Serious infection,^f^ *n* (%)	64 (6.6)	348 (6.6)	0.93	63 (6.6)	80 (8.4)	0.14
Comorbidity index^g^						
Median score (IQR)	1 (0)	1 (0)	0.60	1 (0)	1 (0)	0.12
Score ≥ 2, *n* (%)	197 (21.1)	1029 (20.3)	0.60	191 (20.9)	216 (23.7)	0.16
Line of TNFi therapy			< 0.001^h^			0.69^h^
First, *n* (%)	253 (25.9)	2594 (49.5)		250 (26.3)	236 (24.8)	
Second, *n* (%)	285 (29.2)	1593 (30.4)		276 (29.0)	290 (30.5)	
Third or later, *n* (%)	437 (44.8)	1053 (20.1)		426 (44.7)	426 (44.7)	
Concomitant DMARD use						
Nonbiologic DMARDs (excluding MTX), *n* (%)	146 (15.0)	819 (15.6)	0.60	143 (15.0)	166 (17.4)	0.15
MTX and other nonbiologic DMARDs, *n* (%)	85 (8.7)	661 (12.6)	< 0.001	84 (8.8)	105 (11.0)	0.11
MTX (excluding other nonbiologic DMARDs), *n* (%)	469 (48.0)	2597 (49.6)	0.40	456 (47.9)	450 (47.3)	0.78
Concomitant prednisone use			0.45^h^			0.26^h^
None, *n* (%)	679 (70.1)	3695 (70.9)		661 (69.9)	658 (69.3)	
< 10 mg/day, *n* (%)	197 (20.4)	982 (18.8)		193 (20.4)	179 (18.9)	
≥ 10 mg/day, *n* (%)	92 (9.5)	538 (10.3)		91 (9.6)	112 (11.8)	

*Abbreviations: CZP* Certolizumab pegol, *TNFi* Tumor necrosis factor inhibitor, *PS* Propensity score, *BMI* Body mass index, *RA* Rheumatoid arthritis, *CDAI* Clinical Disease Activity Index, *mHAQ*, Modified Health Assessment Questionnaire (disability index), *COPD* Chronic obstructive pulmonary disease, *NMSC* Nonmelanoma skin cancer, *DMARD* Disease-modifying antirheumatic drug, *MTX* Methotrexate

^a^Race was statistically different between the PS-matched groups (*p* = 0.03), but this was not thought to be clinically significant

^b^Insurance categories were not mutually exclusive; therefore, patients could be captured in more than one category

^c^Pulmonary disease included asthma, COPD, and interstitial lung disease/pulmonary fibrosis

^d^Cardiovascular disease included coronary artery disease, myocardial infarction, coronary heart failure requiring hospitalization, acute coronary syndrome, unstable angina, cardiac revascularization procedure, cardiac arrest, ventricular arrhythmia, and other cardiovascular diseases

^e^Malignancy included lung cancer, breast cancer, melanoma skin cancer, and other cancers

^f^Serious infection was defined as any infection for which the patient was hospitalized and/or received intravenous antibiotics

^g^Comorbidity index was a modified version of the Charlson comorbidity index and corresponded to the sum of scores for current and physician-reported prior comorbid conditions, namely myocardial infarction, congestive heart failure, peripheral vascular disease, cerebrovascular disease (captured as stroke or transient ischemic attack), COPD, history of bleeding and/or peptic ulcer, diabetes mellitus, leukemia, lymphoma, solid tumor cancer (excluding nonmelanoma skin cancer [NMSC]), liver disease, and connective tissue disease (including RA, so all patients had a comorbidity index ≥ 1)

^h^ *p* Values were based on chi-square tests to ascertain if the overall distribution differed significantly between the CZP and other TNFi groups

After PS matching, there were no clinically relevant baseline differences between the CZP and other TNFi groups; line of TNFi therapy and concomitant medication use were also similar (Table 2).

### Observed incidence of AEs in the unmatched population

Average follow-up for each of the AEs can be calculated as the number of PY at risk divided by the number of patients considered. For instance, with SIEs in the CZP group, the average follow-up time was 820/975 = 0.84 PY (approximately 10 months).

**Fig. 2:**
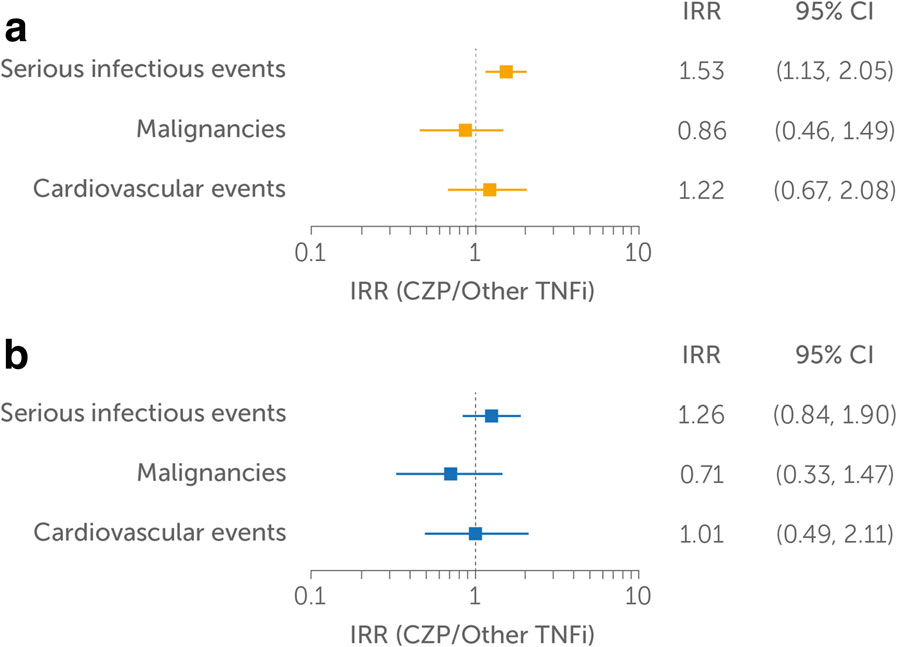
IRRs of SIEs, malignancies, and CVEs between the CZP and other TNFi groups. **a** Before PS matching (all initiators). **b** After PS matching. IRRs were calculated with the CZP group in the numerator and the other TNFi in the denominator. *IRR* Incidence rate ratio, *SIE* Serious infectious event, *CVE* Cardiovascular event, *CZP* Certolizumab pegol, *TNFi* Tumor necrosis factor inhibitor, *PS* Propensity score

Among all initiators, the IR of SIEs was higher in the CZP group than in the other TNFi group (Table 3). The 95% CI of the corresponding IRR excluded unity (IRR 1.53 [1.13, 2.05]), suggesting that CZP was associated with a greater risk of SIEs than other TNFi (Fig. 2a). By contrast, the IRs of malignancies and CVEs did not differ substantially between CZP and other TNFi (Table 3). The 95% CI of the IRRs for malignancies (IRR 0.86 [0.46, 1.49]) and CVEs (IRR 1.22 [0.67, 2.08]) included unity, suggesting no difference in risk between the two groups (Fig. 2a).

**Table 3:** Rates of adverse events of interest before propensity score matching (all initiators)

	CZP (*n* = 975)			Other TNFi (*n* = 5240)		
	PY at risk^a^	Events^b^	IR/100 PY (95% CI)	PY at risk^a^	Events^b^	IR/100 PY (95% CI)
**Serious infectious events**	820	59	**7.20 (5.58, 9.29)**	4566	215	**4.71 (4.12, 5.38)**
Joint/bursa	847	2	0.24 (0.06, 0.94)	4660	16	0.34 (0.21, 0.56)
Cellulitis/skin	841	13	1.55 (0.90, 2.66)	4650	36	0.77 (0.56, 1.07)
Sinusitis	847	2	0.24 (0.06, 0.94)	4660	10	0.21 (0.12, 0.40)
Diverticulitis	847	2	0.24 (0.06, 0.94)	4660	10	0.21 (0.12, 0.40)
Sepsis	848	1	0.12 (0.02, 0.84)	4657	16	0.34 (0.21, 0.56)
Pneumonia	842	15	1.78 (1.07, 2.95)	4641	58	1.25 (0.97, 1.62)
Bronchitis	846	5	0.59 (0.25, 1.42)	4660	11	0.24 (0.13, 0.43)
Gastroenteritis	847	4	0.47 (0.18, 1.26)	4659	14	0.30 (0.18. 0.51)
Meningitis/encephalitis	848	0	0 (NC)	4665	2	0.04 (0.01, 0.17)
Urinary tract infection	842	10	1.19 (0.64, 2.21)	4650	29	0.62 (0.43, 0.90)
Upper respiratory infection	848	2	0.24 (0.06, 0.94)	4660	10	0.21 (0.12, 0.40)
Active tuberculosis^c^	848	0	0 (NC)	4664	2	0.04 (0.01, 0.17)
Other	844	9	1.07 (0.55, 2.05)	4649	36	0.77 (0.56, 1.07)
**Identified serious infectious organism**	839	15	**1.79 (1.08, 2.96)**	4636	65	**1.40 (1.10, 1.79)**
Opportunistic	848	0	0.00 (NC)	4664	2	0.04 (0.01, 0.17)
Nonopportunistic	843	8	0.95 (0.47, 1.90)	4648	30	0.65 (0.45, 0.92)
Unknown	844	7	0.83 (0.40, 1.74)	4648	34	0.73 (0.52, 1.02)
**Malignancies**	841	15	**1.78 (1.08, 2.96)**	4622	96	**2.08 (1.70, 2.54)**
Lymphoma	848	0	0 (NC)	4664	4	0.09 (0.03, 0.23)
Breast cancer	848	1	0.12 (0.02, 0.84)	4660	9	0.19 (0.10, 0.37)
Lung cancer	848	2	0.24 (0.06, 0.94)	4664	3	0.06 (0.02, 0.20)
Skin cancer – melanoma	848	0	0 (NC)	4662	6	0.13 (0.06, 0.29)
Skin cancer – basal/squamous	845	5	0.59 (0.25, 1.42)	4641	49	1.06 (0.80, 1.40)
Other cancer	844	7	0.83 (0.40, 1.74)	4655	26	0.56 (0.38, 0.82)
**Cardiovascular events**	840	17	**2.02 (1.26, 3.26)**	4633	77	**1.66 (1.33, 2.08)**
Myocardial infarction	847	3	0.35 (0.11, 1.10)	4661	11	0.24 (0.13, 0.43)
TIA/stroke	846	5	0.59 (0.25, 1.42)	4650	34	0.73 (0.52, 1.02)
Other cardiovascular event^d^	843	10	1.19 (0.64, 2.20)	4649	43	0.93 (0.69, 1.25)
**Other AEs of interest**						
Anaphylaxis/allergic reaction	845	4	0.47 (0.18, 1.26)	4660	14	0.30 (0.18, 0.51)
Drug-induced SLE	848	0	0 (NC)	4665	0	0 (NC)
Gastrointestinal perforation	848	0	0 (NC)	4663	3	0.06 (0.02, 0.20)
Hepatic event	848	0	0 (NC)	4660	10	0.21 (0.12, 0.40)
Progressive multifocal leukoencephalopathy	848	0	0 (NC)	4665	0	0 (NC)
Other neurological event (with hospitalization)/other demyelinating disease	848	0	0 (NC)	4664	4	0.09 (0.03, 0.23)
Spontaneous serious bleeding	848	0	0 (NC)	4664	5	0.11 (0.04, 0.26)

*Abbreviations: AE* Adverse events, *PS* Propensity score, *CZP* Certolizumab pegol, *TNFi* Tumor necrosis factor inhibitor, *PY* Patient-years, *IR* Incidence rate, *NC* Not calculable, *CVE* Cardiovascular event, *TIA* Transient ischemic attack, *SLE* Systemic lupus erythematosus

^a^Time at risk was measured from drug initiation up to either the occurrence of the first event of interest under that category, 90 days following discontinuation/switch of TNFi, or up to 12 months after drug initiation

^b^Patients may have experienced more than one AE, so the sum of individual conditions may be greater than the total number of first AEs for the overall categories; however, only the time to the first event was considered to calculate the corresponding IRs

^c^Latent tuberculosis was not included

^d^Included congestive heart failure with hospitalization, cardiac revascularization procedure, ventricular arrhythmia, cardiac arrest, acute coronary syndrome, unstable angina, hypertension with hospitalization, peripheral arterial thromboembolic event, urgent peripheral arterial revascularization, peripheral ischemia or gangrene (necrosis), and other CVEs

Cellulitis/skin infection, pneumonia, and urinary tract infection were the most frequently reported SIEs. There were no cases of active tuberculosis among CZP initiators, and there were two cases in the other TNFi group (Table 3). Regarding other AEs of interest, rare cases of anaphylaxis/allergic reaction were reported for both CZP (4 events) and other TNFi (14 events). Patients in the other TNFi group also reported gastrointestinal perforation (three events), hepatic events (ten events), other neurological events (four events), and spontaneous serious bleeding (five events) (Table 3).

### Observed incidence of AEs in the PS-matched population

In contrast with the unmatched population, the IR of SIEs was similar between CZP and other TNFi after PS matching (Table 4). The 95% CI of the IRR included unity (IRR 1.26 [0.84, 1.90]), suggesting that the risk of SIEs was not different between the PS-matched groups (Fig. 2b). Similar to what was seen in the unmatched population, the IRs of malignancies and CVEs in the CZP and other TNFi groups did not differ substantially after PS matching (Table 4). The 95% CI of the IRRs for malignancies (IRR 0.71 [0.33, 1.47]) and CVEs (IRR 1.01 [0.49, 2.11]) included unity, suggesting no difference in risk between the PS-matched groups (Fig. 2b).

**Table 4:** Rates of adverse events of interest after propensity score matching

	CZP (*n* = 952)			Other TNFi (*n* = 952)		
	PY at risk^a^	Events^b^	IR/100 PY (95% CI)	PY at risk^a^	Events^b^	IR/100 PY (95% CI)
**Serious infectious events**	799	57	**7.13 (5.50, 9.25)**	814	46	**5.65 (4.23, 7.54)**
Joint/bursa	825	2	0.24 (0.06, 0.97)	836	3	0.36 (0.12, 1.11)
Cellulitis/skin	820	13	1.59 (0.92, 2.73)	833	9	1.08 (0.56, 2.08)
Sinusitis	826	2	0.24 (0.06, 0.97)	835	2	0.24 (0.06, 0.96)
Diverticulitis	826	2	0.24 (0.06, 0.97)	835	2	0.24 (0.06, 0.96)
Sepsis	826	1	0.12 (0.02, 0.86)	836	3	0.36 (0.12, 1.11)
Pneumonia	821	15	1.83 (1.10, 3.03)	833	9	1.08 (0.56, 2.08)
Bronchitis	825	5	0.61 (0.25, 1.46)	835	2	0.24 (0.06, 0.96)
Gastroenteritis	825	4	0.48 (0.18, 1.29)	834	5	0.60 (0.25, 1.44)
Meningitis/encephalitis	827	0	0 (NC)	837	1	0.12 (0.02, 0.85)
Urinary tract infection	821	10	1.22 (0.66, 2.26)	834	6	0.72 (0.32, 1.60)
Upper respiratory infection	827	2	0.24 (0.06, 0.97)	835	3	0.36 (0.12, 1.11)
Active tuberculosis^c^	827	0	0 (NC)	837	1	0.12 (0.02, 0.85)
Other	823	7	0.85 (0.41, 1.78)	832	9	1.08 (0.56, 2.08)
**Identified serious infection organism**	819	14	**1.71 (1.01, 2.89)**	831	14	**1.69 (1.00, 2.85)**
Opportunistic	827	0	0 (NC)	836	2	0.24 (0.06, 0.96)
Nonopportunistic	822	8	0.97 (0.49, 1.95)	835	4	0.48 (0.18, 1.28)
Unknown	824	6	0.73 (0.33, 1.62)	834	8	0.96 (0.48, 1.92)
**Malignancies**	821	14	**1.71 (1.01, 2.88)**	827	20	**2.42 (1.56, 3.75)**
Lymphoma	827	0	0 (NC)	836	2	0.24 (0.06, 0.96)
Breast cancer	827	1	0.12 (0.02, 0.86)	837	0	0 (NC)
Lung cancer	827	2	0.24 (0.06, 0.97)	837	0	0 (NC)
Skin cancer – melanoma	827	0	0 (NC)	836	2	0.24 (0.06, 0.96)
Skin cancer – basal/squamous	825	4	0.48 (0.18, 1.29)	833	8	0.96 (0.48, 1.92)
Other cancer	823	7	0.85 (0.41, 1.78)	833	8	0.96 (0.48, 1.92)
**Cardiovascular events**	819	17	**2.08 (1.29, 3.34)**	829	17	**2.05 (1.27, 3.30)**
Myocardial infarction	825	3	0.36 (0.12, 1.13)	837	1	0.12 (0.02, 0.85)
TIA/stroke	824	5	0.61 (0.25, 1.46)	832	10	1.20 (0.65, 2.23)
Other cardiovascular event^d^	822	10	1.22 (0.65, 2.26)	833	9	1.08 (0.56, 2.08)
**Other AEs of interest**						
Anaphylaxis/allergic reaction	824	4	0.49 (0.18, 1.29)	835	5	0.60 (0.25, 1.44)
Drug-induced SLE	827	0	0 (NC)	837	0	0 (NC)
Gastrointestinal perforation	827	0	0 (NC)	837	1	0.12 (0.02, 0.85)
Hepatic event	827	0	0 (NC)	834	4	0.48 (0.18, 1.28)
Progressive multifocal leukoencephalopathy	827	0	0 (NC)	837	0	0 (NC)
Other neurological event (with hospitalization)/other demyelinating disease	827	0	0 (NC)	837	0	0 (NC)
Spontaneous serious bleeding	827	0	0 (NC)	837	1	0.12 (0.02, 0.85)

*Abbreviations: AE* Adverse events, *PS* Propensity score, *CZP* Certolizumab pegol, *TNFi* Tumor necrosis factor inhibitor, *PY* Patient-years, *IR* Incidence rate, *NC* Not calculable, *CVE* Cardiovascular event, *TIA* Transient ischemic attack, *SLE* Systemic lupus erythematosus

^a^Time at risk was measured from drug initiation up to either the occurrence of the first event of interest under that category, 90 days following discontinuation/switch of TNFi, or up to 12 months after drug initiation

^b^Patients may have experienced more than one AE, so the sum of individual conditions may be greater than the total number of first AEs for the overall categories; however, only the time to the first event was considered to calculate the corresponding IRs

^c^Latent tuberculosis was not included

^d^Included congestive heart failure with hospitalization, cardiac revascularization procedure, ventricular arrhythmia, cardiac arrest, acute coronary syndrome, unstable angina, hypertension with hospitalization, peripheral arterial thromboembolic event, urgent peripheral arterial revascularization, peripheral ischemia or gangrene (necrosis), and other CVEs

Cellulitis/skin infection, pneumonia, and urinary tract infection were still the most frequent SIEs. The incidence rates of other AEs of interest remained low in the PS-matched groups (Table 4).

## Discussion

When initiating TNFi therapy in RA, rheumatologists need to carefully balance the potential clinical benefits of treatment with the anticipated risks in each individual patient in light of their demographic characteristics, clinical history, and concomitant medications. Excepting a recently completed head-to-head randomized controlled trial (RCT) comparing the efficacy and safety of CZP and adalimumab, in which investigators reported no clinically significant differences between the two drugs [33], there is a paucity of head-to-head RCTs comparing the safety of TNFi drugs in RA. Observational studies therefore provide a valuable source of comparative safety data.

Using data from the Corrona registry, we compared the safety of CZP with other TNFi as a group (golimumab, etanercept, adalimumab, and infliximab). As with any registry study, channeling bias can arise from the fact that clinicians prescribe treatment not at random but based on personal clinical experience and their patients’ clinical characteristics [26]. To overcome this limitation, we assessed safety outcomes in two cohorts: (1) all patients initiating CZP or other TNFi therapy and (2) the subset of patients matched by PS. In the unmatched population, the rate of SIEs was approximately 50% higher in the CZP group than in the other TNFi group. After using the PS-matched model to minimize channeling bias and resolve clinically significant baseline differences between CZP and other TNFi initiators, the risk of SIEs associated with each group was no longer distinguishable.

The contrasting results in the unmatched and PS-matched cohorts highlight the need to consider baseline patient differences when comparing the risk of AEs between TNFi drugs prescribed at different stages in the course of RA. CZP was prescribed later in the line of therapy than other TNFi. Consequently, patients initiating CZP were, on average, older and had longer disease duration, more active disease, and more functional impairment than patients initiating treatment with other TNFi. This probably explains the higher rate of SIEs in the unmatched CZP group because older age, high disease activity, and disability have previously been identified as risk factors for serious infection in RA [9, 34–38].

Meta-analyses of RCTs have demonstrated that the different TNFi drugs available in RA are equally efficacious at reducing disease activity [18]. Therefore, on the basis of clinical efficacy alone, there has been relative equipoise among rheumatologists regarding which therapy to choose as first and subsequent lines of treatment [39]. However, the safety of TNFi therapy, particularly with regard to infection, continues to be debated. The authors of the first published meta-analysis of serious infection in RA detected an association with TNFi treatment [11], but subsequent meta-analyses performed with greater sample sizes have produced discordant results [14, 15, 17, 18, 20]. Similarly, whereas researchers in some observational studies have reported an association between TNFi and the risk of serious infection [10, 13, 19], others did not find a clear link [12, 16, 21]. In a large meta-analysis of RCTs in which authors compared the safety of all five TNFi drugs across multiple diseases, CZP was associated with higher odds of serious infection than adalimumab, etanercept, and golimumab [40]. However, the diseases and patient populations captured in this meta-analysis differed substantially between drugs, and no correction was made for placebo exposure, which makes the results difficult to interpret. It is known that the background risk of SIEs varies greatly across diseases [37, 41], and even within indications it is strongly dependent on study inclusion criteria and baseline patient characteristics [35]. By contrast, a recent head-to-head clinical trial comparing the efficacy and safety of CZP with adalimumab in patients with RA provided direct evidence of a comparable safety profile for the two drugs, including a similar incidence of serious infection [33].

The IRs of SIEs reported in the present study are consistent with rates observed in other real-world patient populations. For example, in a retrospective study of medical and pharmaceutical data from a large U.S. health insurer, the rate of hospitalized infections among patients with RA with no prior biologic use was 4.6/100 PY, whereas for patients switching to a new biologic at baseline, an IR of 7.0/100 PY was reported [35]. In a retrospective analysis of Medicare claims data for patients with RA treated with prior biologics, the crude IRs of hospitalized infection during TNFi treatment ranged between 14.1/100 PY (golimumab) and 17.0/100 PY (infliximab; the IR for CZP was 14.2/100 PY) [42]. A prospective observational study using data from the British Society for Rheumatology Biologics Register – Rheumatoid Arthritis (BSRBR-RA) reported rates of SIEs ranging from 2.8/100 PY in patients aged < 55 years to 8.3/100 PY in patients aged > 75 years [13].

Regardless of PS matching, there were no important differences in the IRs of malignancies and CVEs between CZP and other TNFi initiators. Interpretation of these results must take into account the infrequent nature of these AEs and the fact that patients in this study were followed for a maximum of 12 months, which may have been too short to detect meaningful risk differences between groups.

Evaluating the impact of TNFi on the risk of cancer in RA is a challenging task. Because most types of cancer occur very rarely, RCTs often lack sufficient power to conclusively assess the risk of malignancy, owing to the short duration of follow-up. Furthermore, patients with RA with a history of cancer tend to be excluded from participation in RCTs [43]. Given the latency of cancer and its potential for reactivation over time, it is important to evaluate the risks of TNFi use in patients with a history of this condition. Approximately 5% of Corrona patients included in our study had a history of prior malignancy. Regardless of PS matching, the IRs of malignancies reported here for CZP and other TNFi initiators were comparable to those previously reported in the BSRBR-RA [43, 44] and the Swedish Biologics Registry [45]. Also consistent with these two registries, nonmelanoma skin cancer was one of the most commonly reported malignancies [46, 47].

Cardiovascular disease is one of the main causes of morbidity and premature mortality in patients with RA [3]. This has been attributed to the direct impact of chronic inflammation on the vascular system and to the secondary effects of physical inactivity [48]. By effectively reducing disease activity, TNFi treatment may help to mitigate the cardiovascular risk associated with RA [7, 8]. In this study, traditional cardiovascular risk factors such as BMI, smoking status, blood pressure, history of cardiovascular disease, and prednisone use were similar at drug initiation between the CZP and other TNFi groups. By contrast, RA disease activity, which is also associated with cardiovascular risk [8, 49, 50], was higher in the CZP group. Nevertheless, the IRs of CVEs we report, which corresponded mainly to myocardial infarction and transient ischemic attack (TIA)/stroke, did not differ substantially between the CZP and other TNFi groups, regardless of PS matching.

A limitation of this study was that patients were followed for a maximum of 12 months after initiation of a particular TNFi. Although SIE rates tend to be highest during the first 6–12 months of TNFi exposure [13, 51], this time frame may not have been sufficient to detect differences in the rates of CVEs or malignancies, owing to the comparative infrequency and longer-term nature of these AEs. Despite this limitation, the rates of malignancy we report were consistent with a recent meta-analysis of observational studies where there were no differences among the five TNFi drugs regarding the overall risk of cancer [52]. Similarly, the rates of myocardial infarction and TIA/stroke were comparable to those previously reported for the Corrona cohort [8]. The rarity of other AEs of interest prevented further analyses; their inclusion in this report was meant to provide a more complete picture of the AEs reported by the study population. Overall, although AEs were assessed over a 1-year period only, the findings of this study are consistent with previously published long-term safety analyses of CZP in RA [53].

A key strength of this study is the fact that we compared safety outcomes for TNFi in U.S. clinical practice using data from a large, nationwide cohort of patients with RA. Because patient characteristics and access to biologic drugs can vary substantially between countries, owing to payer and regulatory differences, it is important to obtain clinical evidence directly relevant to practicing rheumatologists in the United States. It has been demonstrated that patients in Corrona share similar demographic and clinical characteristics with Medicare beneficiaries with claims for rheumatology or RA, suggesting that the results we present may be generalizable to the wider RA population in the United States [54]. Furthermore, we used PS matching to control for baseline patient characteristics that might have influenced the decision to treat with CZP or other TNFi, thereby helping to minimize channeling bias and allowing for a more accurate comparison of the risk of AEs associated with these therapies.

## Conclusions

In a PS-matched cohort of patients with moderate to severe RA enrolled in the Corrona registry, there were no differences in the 1-year risk of SIEs, malignancies, and CVEs between patients treated with CZP and those treated with other TNFi.

## Additional file

Additional file 1: Modified Charlson comorbidity index. **Table S1.** Other AEs of interest. (PDF 246 kb)

## Abbreviations

AE: Adverse event
BMI: Body mass index
BSRBR-RA: British Society for Rheumatology Biologics Register – Rheumatoid Arthritis
CDAI: Clinical Disease Activity Index
COPD: Chronic obstructive pulmonary disease
Corrona: Consortium of Rheumatology Researchers of North America
CVE: Cardiovascular event
CZP: Certolizumab pegol
DMARD: Disease-modifying antirheumatic drug
IR: Incidence rate
IRB: Institutional review board
IRR: Incidence rate ratio
IV: Intravenous
mHAQ: Modified Health Assessment Questionnaire
MRSA: Methicillin-resistant *Staphylococcus aureus*
MTX: Methotrexate
NC: Not calculable
NMSC: Nonmelanoma skin cancer
PS: Propensity score
PY: Patient-years
RA: Rheumatoid arthritis
RCT: Randomized controlled trial
SIE: Serious infectious event
SLE: Systemic lupus erythematosus
TIA: Transient ischemic attack
TNF: Tumor necrosis factor
