# Supplementary material for: One-year risk of serious infection in patients treated with certolizumab pegol as compared with other TNF inhibitors in a real-world setting: data from a national U.S. rheumatoid arthritis registry
Source: Arthritis Res Ther. 2018 Jan 2;20:2. doi: 10.1186/s13075-017-1496-5 (PMC5795286; doi:10.1186/s13075-017-1496-5)
Supplement: Additional file 1: — Modified Charlson comorbidity index. Table S1. Other AEs of interest. (PDF 246 kb) [file 13075_2017_1496_MOESM1_ESM.pdf]

One-Year Risk of Serious Infection in Patients Treated with Certolizumab Pegol as Compared with Other TNF Inhibitors in a Real-World Setting: Data from a National US Rheumatoid Arthritis Registry

Leslie R. Harrold, Heather J. Litman, Katherine C. Saunders, Kimberly J. Dandreo, Bernice Gershenson, Jeffrey D. Greenberg, Robert Low, Jeffrey Stark, Robert Suruki, Srihari Jaganathan, Joel M. Kremer, Mohamed Yassine

Additional File

ADDITIONAL FILE

Supplementary Table S1. Other AEs of interest

AE category	Physician-reported AEs included in the analysis
**Anaphylaxis/allergic reaction**	Any severe or anaphylactic reaction to a TNFi or systemic therapy for the disease under study
**Drug-induced SLE**	Drug-induced SLE was reported via Provider Questionnaires in cases where patients were diagnosed with SLE by a treating physician. The investigator/treating physician used appropriate clinical judgement in determining whether the patient was suspected to have experienced drug-induced SLE.
**Gastrointestinal perforation**	Upper or lower gastrointestinal perforation
**Hepatic events**	Hepatic events or conditions that were considered serious[a] and/or required biopsy
**Progressive multifocal leukoencephalopathy**	Physician-confirmed diagnoses of John Cunningham virus infection resulting in progressive multifocal leukoencephalopathy
**Other neurological events with hospitalization and/or other demyelinating disease**	Demyelinating disease (eg. multiple sclerosis), and other confirmed, serious[a] and/or medically important neurologic conditions in the opinion of the investigator (eg. amyotrophic lateral sclerosis, Guillain-Barre syndrome)
**Spontaneous serious bleeding**	Hemorrhage requiring hospitalization or otherwise meeting serious AE criteria[a]

[a]Serious AE (SAE) was any event occurring in a patient enrolled in the Corrona rheumatoid arthritis registry during the course of study participation that resulted in one or more of the following (based on US Food and Drug Administration definition of SAEs): hospitalization (initial or prolonged); death; immediately life threatening (emergency intervention required to prevent death); significant or persistent disability; congenital anomaly or birth defect; other serious event (important medical events) that does not fit the other outcomes, but that may jeopardize the patient and may require medical or surgical intervention (treatment) to prevent one of the other outcomes. AE: adverse event; TNFi: tumor necrosis factor inhibitor; SLE: systemic lupus erythematosus.

Modified Charlson comorbidity index

Patients' comorbidity scores were calculated using a version of the Charlson comorbidity index,[32] modified to encompass current and prior physician-diagnosed comorbidities captured in the Corrona database. These were myocardial infarction, congestive heart failure, peripheral vascular disease, cerebrovascular disease (captured as stroke or transient ischemic attack), chronic obstructive pulmonary disease, history of peptic and/or gastrointestinal bleeding ulcer (not just peptic ulcer, as proposed by the original Charlson comorbidity index[32]), diabetes mellitus, leukemia, lymphoma, solid tumor cancer (excluding non-melanoma skin cancer), liver disease, and connective tissue disease. Corrona does not capture the following comorbidities: dementia, kidney disease, hemiplegia, and human immunodeficiency virus/acquired immunodeficiency syndrome (HIV/AIDS).
